# Psychometric validation of the Chinese version of the Scratch Intensity and Impact Scale (SIIS) in patients with chronic pruritus

**DOI:** 10.3389/fpubh.2026.1836131

**Published:** 2026-08-17

**Authors:** Jian Sun, Yiru Chang, Lun He, Xue Tian

**Affiliations:** Department of Dermatology and Venereology, The First Affiliated Hospital of Jinzhou Medical University, Jinzhou, Liaoning, China

**Keywords:** chronic pruritus, health-related quality of life, patient-reported outcome measures, psychometric validation, Scratch Intensity and Impact Scale

## Abstract

**Background:**

Chronic pruritus poses a substantial burden on patients, leading to physical discomfort, emotional distress, and psychosocial dysfunction. The Scratch Intensity and Impact Scale (SIIS) is a patient-reported outcome (PRO) measure designed to assess both scratching intensity and its effects on health-related quality of life (HRQoL). However, its psychometric properties have not been validated in a Chinese population. This study aimed to culturally adapt and psychometrically validate the SIIS for Chinese patients with chronic pruritus.

**Methods:**

The original SIIS was translated into Chinese following a standardized forward-backward translation procedure. In a cross-sectional study, 358 adult patients with chronic pruritus were recruited from Northeast China. Construct validity was assessed by randomly splitting the sample for exploratory factor analysis (EFA; *n* = 179) with oblique rotation and confirmatory factor analysis (CFA; *n* = 179). Reliability was evaluated through internal consistency (Cronbach’s *α* and McDonald’s *ω*), split-half reliability, and two-week test–retest reliability. Content validity was examined using content validity indices.

**Results:**

The 13-item Chinese version of the SIIS exhibited a stable two-factor structure, explaining 84.378% of the total variance. Sampling adequacy was confirmed (KMO = 0.948), and Bartlett’s test indicated suitability for factor analysis (χ^2^ = 6798.064, *p* < 0.001). CFA demonstrated acceptable model fit: chi-square degree of freedom (*χ*^2^/df) = 2.094, root mean square error of approximation (RMSEA) = 0.078, standardized root mean square residual (SRMR) = 0.0188. Comparative Fit Index (CFI) = 0.985, Tucker–Lewis Index (TLI) = 0.981, Normed Fit Index (NFI) = 0.972, and Incremental Fit Index (IFI) = 0.985. Content validity was strong, with a scale-level content validity index (S-CVI) of 0.96 and item-level indices (I-CVI) ranging from 0.83 to 1.00. The scale also showed excellent reliability, including high internal consistency (Cronbach’s *α* = 0.971, McDonald’s *ω* = 0.969), split-half reliability (Spearman–Brown coefficient = 0.904), and strong test–retest stability over 2 weeks (*r* = 0.958).

**Conclusion:**

The Chinese version of the SIIS demonstrates strong psychometric properties, confirming its reliability and validity as a PRO instrument for evaluating scratching intensity and HRQoL impact. In this population, it shows promise as a PRO instrument for adult patients with chronic pruritus due to AD or psoriasis in dermatology settings, pending further validation in broader populations.

## Introduction

Chronic pruritus (CP), defined as persistent itching lasting more than 6 weeks, is a highly prevalent and debilitating symptom that affects the vast majority (70–100%) of patients with inflammatory skin diseases such as atopic dermatitis (AD) and psoriasis (1–3). In China, the burden of these conditions is substantial. AD affects 7–10% of adults, a prevalence notably higher than in many Western populations (4), while psoriasis, though less prevalent (0.47–0.59%), still impacts a significant number of individuals (5, 6). The severe pruritus characteristic of these conditions frequently initiates a self-perpetuating vicious cycle of scratching and sleep disruption (e.g., insomnia, fatigue), which serves as a primary driver of compromised health-related quality of life (HRQoL) and overall health status (7, 8). Scratching constitutes a core behavioral component of this cycle. While providing transient relief, it paradoxically perpetuates the itch-scratch cycle, exacerbates skin damage through excoriation and lichenification, and intensifies the subjective experience of pruritus, thereby amplifying the overall disease burden. The precise psychophysiological mechanisms intertwining pruritus, scratching, and sleep disruption, however, remain inadequately elucidated (9, 10).

Understanding this vicious cycle is critical, as it acts as a primary driver of the profound disease burden and substantially impaired quality of life associated with chronic itch. The consequences extend beyond the physical domain, encompassing significant emotional distress (e.g., anxiety, frustration), psychosocial impairment (e.g., social withdrawal, stigmatization), and substantial economic costs related to healthcare utilization and lost productivity (11–13). Given these multifaceted burdens, there is an urgent need to accurately assess not only itch severity but also, more holistically, the behavioral response (scratching) and its downstream impacts to inform effective, patient-centered therapeutic strategies. This necessitates a measurement framework that prioritizes the patient’s subjective experience. In contrast, conventional clinical assessments and clinician-reported outcome (ClinRO) measures focus primarily on documenting objective signs such as erythema, lichenification, and excoriations. These tools are inherently limited in their ability to capture the internal, subjective burden and lived experience of the itch-scratch cycle, which ultimately defines a patient’s quality of life (14). This well-documented disconnect between clinician-observed signs and patient-reported suffering is a central challenge in managing chronic skin conditions.

Consequently, rigorously validated patient-reported outcome (PRO) measures are indispensable for capturing this subjective experience and elucidating the complex, individualized interplay between itch sensation, scratching behavior, sleep quality, and overall HRQoL. PRO instruments are founded on the principle that the patient is the optimal source of information about their own symptoms and the impact of those symptoms on function and well-being. Their rigorous development and validation are therefore central to the field of outcomes research focused on HRQoL. Current instruments for assessing chronic pruritus suffer from several key shortcomings: many lack specificity for scratching behavior and its associated burden, others exhibit suboptimal psychometric properties, and some are limited to single-item assessments. For instance, clinician-rated indices such as the Eczema Area and Severity Index (EASI) and SCORing Atopic Dermatitis (SCORAD) focus on skin signs rather than the patient’s experience of scratching or its impact on sleep and quality of life (15, 16). Furthermore, while objective monitoring technologies (e.g., accelerometry-based wrist monitors, acoustic sensors) offer promising avenues for quantifying scratching behavior in research settings (17–19), their application in routine clinical practice is often constrained by limited validation data, high cost, technical complexity, and patient acceptability barriers. This landscape underscores a persistent critical gap: the absence of a rigorously validated, multidimensional PRO measure specifically designed to capture both the intensity of scratching behavior and its direct, multifaceted impact on HRQoL from the patient’s perspective.

To address this gap, the Scratch Intensity and Impact Scale (SIIS) was developed as a patient-reported outcome (PRO) instrument specifically designed to quantify the dual dimensions of chronic pruritus burden: scratching intensity and its impact on health-related quality of life (HRQoL) (20). However, its application in the Chinese context has been limited by the absence of a linguistically and culturally adapted version. Therefore, this study was designed with two primary objectives: (1) to perform the cross-cultural adaptation of the SIIS into Chinese, and (2) to rigorously evaluate the psychometric properties of the adapted Chinese version (SIIS-C) in patients with chronic pruritus. The psychometric evaluation will encompass internal consistency, test–retest reliability, construct validity (using both exploratory and confirmatory factor analyses), and convergent validity with the 5-D itch scale (21). The successful validation of this instrument will provide a critical, patient-centered tool for both clinical management and outcome research in chronic pruritus within the Chinese healthcare context.

## Methods

### Translation and cross-cultural adaptation

Following authorization by its original developer, Professor Heckman, the SIIS was translated and cross-culturally adapted according to Brislin’s model (22) and our institution’s established validation protocol (23, 24).

### Forward translation and synthesis

Two independent bilingual translators (one with a medical and one with a psychological background) produced initial forward translations. A multidisciplinary committee of researchers and linguists then reviewed these translations. Through an iterative consensus process, a single preliminary Chinese version was synthesized, prioritizing conceptual equivalence over literal translation and minimizing individual translator bias.

### Back-translation and expert review

Two additional bilingual translators, naive to the original SIIS, independently back-translated the synthesized Chinese version. The research team then convened to compare all versions (original, synthesized, back-translated), discussing and resolving any semantic, conceptual, or experiential discrepancies to ensure fidelity to the original instrument’s intent.

### Cognitive debriefing and finalization

The pre-final Chinese version of the SIIS was evaluated in a pilot cognitive debriefing study. To assess face validity and cognitive equivalence, a convenience sample of 20 adult Chinese patients diagnosed with chronic pruritus was recruited for cognitive debriefing. Participants completed the pre-final version and subsequently provided structured feedback on item clarity, comprehensibility, and overall acceptability through a semi-structured interview. The pilot results confirmed excellent readability and face validity, with no items identified as confusing or problematic. The average completion time was approximately 3 min. Consequently, the pre-final version was ratified as the final SIIS-Chinese version (SIIS-C) for use in the subsequent large-scale psychometric validation study.

### Study design and participants

This cross-sectional validation study enrolled adult patients with chronic pruritus at the First Affiliated Hospital of Jinzhou Medical University, China. Eligible participants were Chinese-speaking adults aged 18–70 years with a physician-confirmed diagnosis of either atopic dermatitis (U. K. Working Party’s Diagnostic Criteria) or psoriasis (International Psoriasis Council criteria). A target sample size of 5–10 participants per item was set, consistent with established methodological precedents for scale validation at our institution (25). This yielded a final analytic cohort of 358 participants who completed all baseline measures. The study received ethical approval from the Institutional Review Board of Jinzhou Medical University (Approval ID: JZMULL2023106) in accordance with the Declaration of Helsinki. Written informed consent was obtained from all participants following a detailed study briefing. All participants completed the SIIS-C and the 5-D itch scale at baseline. A randomly selected subgroup (*n* = 50) then repeated both assessments after 2 weeks to evaluate test–retest reliability. Data collected at baseline included socio-demographic characteristics (age, gender, education, employment, marital status, household income) and clinical information (specific dermatologic diagnosis).

### Instruments

#### Sociodemographic and clinical characteristics questionnaire

Sociodemographic and clinical characteristics were collected using a study-specific questionnaire, which captured age, gender, educational attainment, employment and marital status, household income, disease classification, and relevant clinical features.

#### Original scratch intensity and impact scale (SIIS)

The original SIIS was developed through a multi-step process incorporating literature synthesis, patient interviews, and expert consensus involving dermatologists, psychologists, and outcomes researchers (20). This 13-item instrument comprises two subscales: a 5-item Scratching Intensity subscale and an 8-item Impact on HRQoL subscale. All items employ a 5-point Likert scale (0 = “Never” to 4 = “Always”), yielding a total score range of 0–52, where higher scores denote greater scratching severity and HRQoL impairment. The scale has demonstrated robust psychometric properties. The original validation reported excellent internal consistency (Cronbach’s *α* = 0.93 for the total scale, 0.91 for the intensity subscale, and 0.85 for the impact subscale) and good construct validity, as evidenced by a significant correlation with the 5-D Pruritus Scale (*r* = 0.59) (20).

### The 5-D pruritus scale

The 5-D Pruritus Scale is a 5-item self-reported measure originally developed by Elman et al. (21). It assesses the multidimensional burden of pruritus over the preceding 2 weeks across five domains: Degree, Duration, Direction, Disability, and Distribution. Each domain is individually scored, yielding a total score between 5 and 25; higher scores represent more severe pruritus. The scale is a well-validated and reliable measure widely used in both clinical and research settings (21). In this study, it was utilized to examine convergent validity, and its test–retest reliability was assessed over a two-week period.

### Statistical analysis

Data analysis was performed using IBM SPSS Statistics (version 27.0) for descriptive analyses and IBM AMOS (version 24.0) for structural equation modeling. Descriptive statistics (means and standard deviations) were calculated for all items. Univariate normality was evaluated by assessing skewness and kurtosis indices; absolute values below 1 and 2, respectively, were taken to indicate excellent normality (26). The SIIS employs a 5-point Likert scale. Although Likert-scale data are fundamentally ordinal, methodological research has demonstrated that treating such data as continuous is acceptable when (a) the number of response categories is ≥ 5, (b) item response distributions are approximately symmetric, and (c) sample sizes are adequate (27, 28). All SIIS items satisfied these conditions (see Table 1 for skewness and kurtosis). For CFA, we used robust maximum likelihood (MLR) estimation, which provides standard errors and fit statistics robust to non-normality (27, 29, 30).

**Table 1:** Mean (SD) scores with skewness and kurtosis figures (*N =* 358).

Items	Mean (SD)	Skewness	Kurtosis
1 I scratch a lot	2.09(0.72)	0.441	−0.246
2 I break the skin while scratching	2.00(0.93)	0.448	−0.337
3 I get marks on my skin from scratching	1.73(0.78)	0.462	−0.314
4 Feeling stressed/anxious makes me scratch more	2.53(1.05)	0.203	−0.670
5 I try to hide my scratching from others	2.44(0.86)	0.232	−0.641
6 My scratching impacts my work	2.05(1.36)	0.354	−0.589
7 My scratching impacts my daily activities	2.15(0.83)	0.341	−0.417
8 I continue to scratch even when I do not feel itchy	1.59(0.67)	0.537	−0.211
9 I have to make a big effort to control my scratching	1.93(0.89)	0.516	−0.193
10 I do a lot of different things to try to control myscratching	2.49(0.97)	0.295	−0.592
11The quality of my sleep is affected by my scratching	2.29(1.06)	0.286	−0.678
12 The amount of time I sleep is affected by my scratching	2.31(1.12)	0.279	−0.467
13Feeling tired or not being able to sleep makes my scratching worse	2.21(1.25)	0.169	−0.538

Where theoretically justified by content overlap and empirically supported by modification indices, correlated residuals were permitted within and, where substantively meaningful, across factors. All such correlations were theoretically interpretable.

Exploratory factor analysis (EFA) was conducted using Principal Axis Factoring (PAF) as the extraction method to identify potential latent constructs (31). Confirmatory factor analysis (CFA) was additionally performed to validate the factor structure (32). Criteria for the recommended indices included (33–35): (a) Chi-squared divided by the degrees of freedom (*χ*^2^/df) ≤ 3; (b) root mean squared error of approximation (RMSEA) < 0.08; (c) standardized root mean square residual (SRMR) < 0.08; (d) comparative fit index (CFI), Tucker–Lewis index (TLI), normed fit index (NFI), and incremental fit index (IFI) > 0.90. Convergent validity was considered satisfactory if the average variance extracted (AVE) value exceeded 0.50 and the composite reliability (CR) value surpassed 0.70 (36, 37). Convergent validity was assessed by examining the Pearson correlation between the SIIS total score and the 5-D Itch Scale total score, with a correlation coefficient ≥ 0.50 considered evidence of adequate convergent validity. Discriminant validity was established by meeting two criteria: (1) the square root of the average variance extracted (AVE) for each construct exceeded the inter-construct correlation satisfying the Fornell-Larcker criterion (38); and (2) all Heterotrait–Monotrait (HTMT) ratios of correlations were below the threshold of 0.85 (39). Internal consistency was estimated using Cronbach’s alpha, McDonald’s omega, and split-half reliability, with conventional thresholds applied (*α* ≥ 0.70; *ω* ≥ 0.80) (40, 41). Temporal stability was examined via a two-week test–retest design in a randomly selected subsample of 50 participants. Participants were asked at the second assessment: ‘Compared to 2 weeks ago, how would you rate the overall severity of your itching?’ with response options: (1) Much worse, (2) Somewhat worse, (3) About the same (stable), (4) Somewhat better, (5) Much better. Only participants who reported ‘About the same’ (stable) were included in the test–retest reliability analysis. Test–retest reliability was assessed using the intraclass correlation coefficient (ICC) based on a two-way mixed-effects model for absolute agreement with single measures, which is the recommended approach for continuous PRO measures. An ICC value ≥ 0.70 was considered acceptable (42).

## Results

### Socio-demographic characteristics of participants

A total of 387 individuals were initially recruited. After excluding 29 participants with incomplete data (7.5%), the final analytical sample comprised 358 respondents, including 183 patients with atopic dermatitis and 175 with psoriasis. As detailed in Table 2, the mean age was 40.2 years (SD = 12.07), and 51.1% of participants were male. The majority were married (52.7%), employed full-time (61.7%), and had completed at least secondary education (79.1%). The most frequently reported annual household income was below $20,000 (43.9%).

**Table 2:** Socio-demographic characteristics of participants (*N* = 358).

Characteristics	Groups	Psychometric sample		
		Psoriasis (*n* = 175)	AD (*n* = 183)	Total (*n* = 358)
Age(years) (mean ± SD)		41.6 (12.53)	38.5 (11.76)	40.2 (12.07)
Disease categories				
	Psoriasis	175(100%)	0(0)	175(48.9%)
	AD	0(0)	183(100%)	183(51.1%)
Gender				
	Male	101(57.7%)	82(44.9%)	183(51.1%)
	Female	74(42.3%)	101(55.1%)	175(48.9%)
Educational background				
	Primary or less	34(19.4%)	37(20.2%)	75(20.9%)
	Secondary or higher	141(80.6%)	146(79.8%)	283(79.1%)
Employment				
	Full time	118(67.4%)	103(56.3%)	221(61.7%)
	Non-full-time	57(32.6%)	80(43.7%)	137(38.3%)
Marital status				
	Single	37(21.1%)	67(36.6%)	104(29.1%)
	Married	97(55.4%)	91(49.7%)	189(52.7%)
	Separated or divorced	41(23.5%)	25(13.7%)	65(18.2%)
Household income				
	<$20,000	79(45.1%)	76(41.5%)	157(43.9%)
	$20,000–$40,000	62(35.4%)	72(39.3%)	133(37.1%)
	>$40,000	34(19.5%)	35(19.2%)	68(19.0%)

AD, atopic dermatitis.

All values are in % (*n*), unless otherwise indicated.

### Descriptive statistics

Item-level descriptive statistics for the Chinese version of the SIIS are presented in Table 1. The total scale score averaged 28.81 (SD = 9.38). All skewness and kurtosis values fell within acceptable ranges, confirming the appropriateness of parametric tests. Notably, a clinically meaningful burden (item mean > 2) was reported for the majority of items (10 of 13, 76.9%), indicating a moderate-to-severe overall impact of chronic pruritus. The items reflecting the greatest impairment pertained to the HRQoL impact dimension, particularly those addressing stress-induced scratching, the use of diverse coping strategies, and attempts to conceal scratching from others. Conversely, items measuring the direct behavioral intensity of scratching [e.g., effort to control, scratching in the absence of itch, and physical sequelae such as marks, frequency, and skin breakdown consistently reflected a moderate level of severity (mean range: 1–2)].

The total SIIS score in this study ranged from 12 to 48. The floor effect (proportion scoring the minimum possible total score of 0) was 0%, and the ceiling effect (proportion scoring the maximum possible total score of 52) was 0%. Both values were well below the commonly accepted threshold of 15%, indicating no substantial floor or ceiling effects, and supporting the scale’s ability to discriminate across different levels of scratching severity.

#### Item analysis

Results of the item analysis are presented in Table 3. All 13 items showed strong corrected item-total correlations (range: 0.834–0.884), exceeding the recommended threshold of 0.3 (43). The scale demonstrated excellent internal consistency, with a Cronbach’s alpha of 0.971. The high reliability was further evidenced by the fact that deleting any item would lower the overall alpha coefficient, confirming that every item contributed positively to the scale’s homogeneity. Therefore, all 13 items were retained for subsequent analyses.

**Table 3:** Item-total correlations for the Chinese SIIS (*N* = 358).

Item	*r*	Cronbach alpha if the item was deleted	Corrected item-total correlation
1	0.876	0.968	0.853
2	0.868	0.968	0.857
3	0.863	0.968	0.853
4	0.841	0.969	0.849
5	0.843	0.969	0.851
6	0.835	0.969	0.842
7	0.856	0.968	0.863
8	0.879	0.968	0.867
9	0.871	0.968	0.858
10	0.871	0.968	0.878
11	0.870	0.968	0.876
12	0.878	0.968	0.884
13	0.827	0.969	0.834

#### Validity analysis

##### Construct validity

Construct validity was evaluated using an independent split-sample validation design. The total sample (*N* = 358) was randomly divided into two independent subsamples.

Exploratory factor analysis (EFA) with oblique rotation was conducted on Subsample.

1 (*n* = 179) to identify the latent factor structure. The model derived from the EFA was subsequently tested via confirmatory factor analysis (CFA) on Subsample 2 (*n* = 179) to assess its goodness-of-fit. All analytical procedures and fit criteria adhered to established protocols, consistent with prior validation studies of Chinese populations conducted at our institution (44, 45).

#### Exploratory factor analysis (EFA)

Exploratory factor analysis (EFA) was conducted on Subsample 1 (*n* = 179) using Principal Axis Factoring (PAF) with Promax (oblique) rotation. The data were highly suitable for factor analysis, as indicated by an excellent Kaiser–Meyer–Olkin (KMO) measure of 0.948 and a significant Bartlett’s test of sphericity (*χ*^2^ = 6798.064, *p* < 0.001). The analysis revealed a clear two-factor solution. The oblique rotation revealed a substantial correlation between the two factors (*r* = 0.722, *p* < 0.001), empirically confirming that the factors are not independent and justifying our choice of oblique rotation. Both factors had eigenvalues greater than 1 and together accounted for 84.378% of the total variance (Factor 1: 73.981%; Factor 2: 10.396%). All 13 items demonstrated high factor loadings, ranging from 0.805 to 0.889, with each item loading predominantly on its intended theoretical factor. Complete results are detailed in Table 4.

**Table 4:** Factor loadings of the exploratory factor analysis with 13 items (*n_1_* = 179).

SIIS Item number	Component	
	Scratching intensity	Impact of scratching on QOL
1	0.873	
2	0.878	
3	0.871	
4		0.889
5		0.874
6		0.821
7		0.824
8	0.816	
9	0.841	
10		0.857
11		0.857
12		0.866
13		0.805

**Figure 1:**
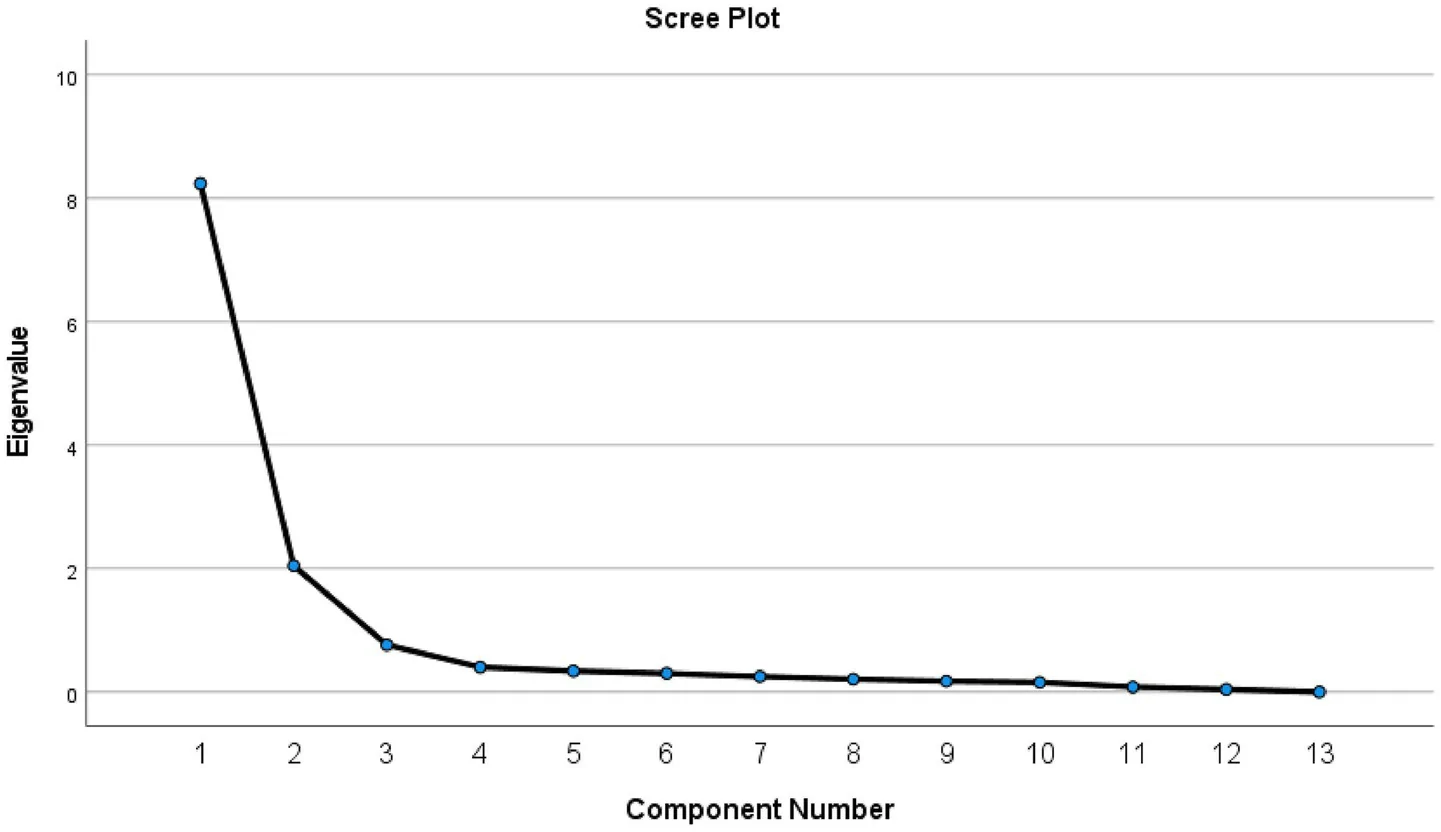
Scree plot from the EFA of the Chinese SIIS (*n* = 179).

The two-factor solution was further corroborated by the scree plot, where a clear inflection point was evident after the second component (Figure 1).

#### Confirmatory factor analysis (CFA)

The two-factor structure identified in the EFA was subsequently subjected to CFA using the independent Subsample 2 (*n* = 179). The model exhibited an excellent fit to the data, with all key indices surpassing established benchmarks for good model fit: *χ*^2^/df = 2.094, RMSEA = 0.078, SRMR = 0.0188, CFI = 0.985, TLI = 0.981, NFI = 0.972, and IFI = 0.985. The CFA results are shown in Table 5.

**Table 5:** CFA fit indices for the SIIS two-factor model of SIIS (*n* = 179).

Fit Index	Criterion	MLR	WLSMV
*χ*^2^/df	<3.0	2.094	2.315
RMSEA	<0.08	0.078	0.079
SRMR	<0.08	0.0188	0.0223
CFI	>0.90	0.985	0.983
TLI	>0.90	0.981	0.979
NFI	>0.90	0.972	0.969
IFI	>0.90	0.985	0.982

*χ*^2^, chi-square; df, degrees of freedom; RMSEA, root mean square error of approximation; SRMR, standardized root mean residual; CFI, comparative fit index; TLI, Tucker–Lewis index; NFI, normed fit index; IFI, incremental fit index.

**Figure 2:**
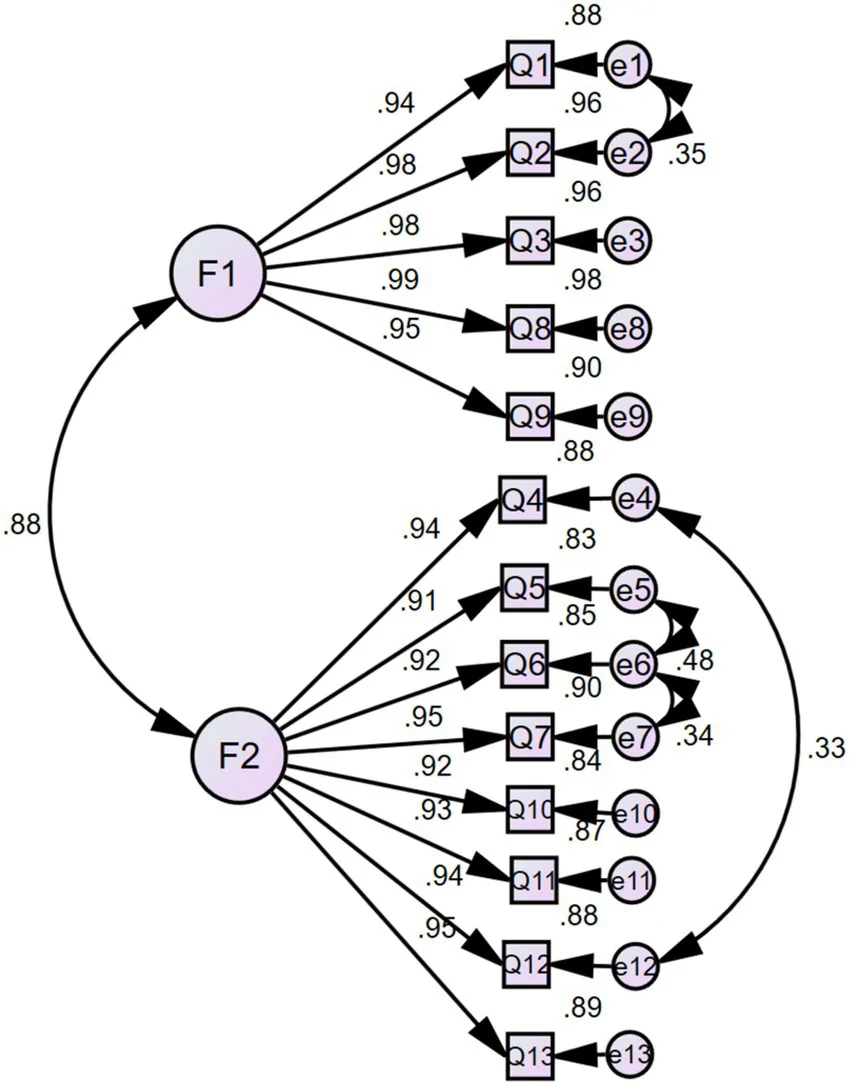
*S*tandardized two-factor model of the Chinese version of the SIIS from the CFA (*n* = 179). F1: scratching intensity (5 items); F2: impact on HRQOL (8 items).

Furthermore, Figure 2 displays the factor loadings of the SIIS items. All standardized factor loadings were exceptionally high, ranging from 0.91 to 0.99 (all *p* < 0.01), substantially above the recommended 0.70 threshold. Collectively, these results provide strong evidence for the robust construct validity of the two-factor model.

In the CFA model, correlated residuals were specified between four item pairs based on content overlap and modification indices: e1–e2 (scratching frequency and severity), e4–e12 (psychological distress and sleep disturbance), e5–e6 (social concealment and work impairment), and e6–e7 (work and general daily activity impairment).

To further validate the robustness of our factor structure given the ordinal nature of the Likert-scale items, we re-estimated the confirmatory factor model using WLSMV (Weighted Least Squares with Mean and Variance adjustment) estimation with a polychoric correlation matrix, which is specifically designed for ordinal data. The WLSMV model demonstrated excellent fit: *χ*^2^/df = 2.315, RMSEA = 0.079, SRMR = 0.022, CFI = 0.983, TLI = 0.979, NFI = 0.969, IFI = 0.982. These results were highly consistent with our primary MLR-based fit indices (*χ*^2^/df = 2.094, RMSEA = 0.078, SRMR = 0.0188, CFI = 0.985, TLI = 0.981, NFI = 0.972, IFI = 0.985), with all differences falling well within acceptable ranges. This consistency confirms that the two-factor structure of the SIIS is robust and not an artifact of the estimation method employed.

### Convergent validity and discriminant validity

The construct validity of the scale was further substantiated through assessments of convergent and discriminant validity (Table 6). Convergent validity was strongly supported. Both composite reliability (CR: 0.982–0.987) and average variance extracted (AVE: 0.872–0.939) substantially exceeded their respective recommended thresholds (CR > 0.70, AVE > 0.50). This was further reinforced by the high maximum reliability coefficients (MaxR(H) > 0.95) for each factor. Discriminant validity was assessed using established criteria: (a) the square root of the average variance extracted (AVE) for each construct (F1 = 0.969, F2 = 0.934) exceeded the inter-construct correlation (Fornell-Larcker criterion); and (b) the Heterotrait–Monotrait ratios of correlations (HTMT = 0.806) were below the threshold of 0.85.

**Table 6:** Convergent validity of the Chinese SIIS.

Classify	CR	AVE	MaxR(H)	√AVE	MSV	HTMT analysis	
						F1	F2
F1	0.987	0.939	0.987	0.969	0.778	–	0.806
F2	0.982	0.872	0.981	0.934	0.778	0.806	–

CR, Composite Reliability; AVE, Average Variance Extracted; Maxr(H), Maximum Reliability;√AVE, the square root of the average variance extracted (AVE); MSV, Maximum Shared Variance; HTMT, Heterotrait–Monotrait Ratio.

### Content validity

Content validity was evaluated by an expert panel comprising four physicians, a psychologist, and a psychometrician (*n* = 6). All items demonstrated strong relevance, with item-level content validity indices (I-CVI) ranging from 0.83 to 1.00. The scale-level content validity index, calculated as the average of all I-CVIs (S-CVI/Ave), was 0.96, exceeding the recommended benchmark of 0.90 (46).

#### Convergent validity with the 5-D itch scale

Convergent validity was assessed by correlating the total score of the Chinese SIIS with that of the 5-D itch scale, a well-validated multidimensional measure of pruritus.

The data for both scales met the assumption of normality (all *p* > 0.05), supporting the use of Pearson’s correlation. A significant positive correlation of moderate-to-strong magnitude was found between the two scales (*r* = 0.591, *p* < 0.001). This moderate-to-strong correlation provides empirical evidence for the convergent validity of the Chinese SIIS.

These results are detailed in Table 7.

**Table 7:** Pearson correlations between the Chinese version of SIIS and 5-D Pruritus Scale.

Classify	SIIS	F1	F2
F1	0.905**	–	–
F2	0.960**	0.722**	–
5-D Pruritus Scale	0.591**	0.362**	0.384**

**Significant correlation at the 0.01 level (two-sided).

#### Reliability analysis

##### Internal consistency reliability

Reliability coefficients for the full scale and its two subscales are presented in Table 8. The full scale demonstrated excellent internal consistency, with an overall Cronbach’s *α* of 0.971. Both subscales also showed high consistency (*α* = 0.980 and 0.962). These findings were further corroborated by McDonald’s omega coefficients (*ω* total = 0.969; *ω* Factor 1 = 0.980; *ω* Factor 2 = 0.961), with all values exceeding the recommended benchmark of 0.70. Additionally, the scale exhibited good split-half reliability (*r* = 0.904). These results indicate high internal consistency reliability for the Chinese SIIS.

**Table 8:** Coefficient of Chinese version of SIIS.

Classify	Cronbach alpha coefficient	McDonald’s Omega coefficient	Split-half reliability
The total sample	0.971	0.969	0.904
F1	0.980	0.980	0.947
F2	0.962	0.961	0.964

##### Test–retest reliability

To assess temporal stability, a randomly selected subsample (*n* = 50) completed the Chinese SIIS twice over a two-week interval. To ensure that score variation reflected measurement stability rather than true clinical change, the analysis was restricted to participants who self-reported stable symptoms at the second assessment. Test–retest reliability was assessed in a randomly selected subsample of 50 participants who completed the SIIS twice over a two-week interval. The ICC was 0.958 (95% CI: 0.928–0.976), indicating excellent test–retest reliability of the Chinese SIIS over a two-week period.

## Discussion

Chronic pruritus is a debilitating symptom that significantly impairs health-related quality of life (HRQoL) through sleep disruption, fatigue, and psychosocial distress across multiple dermatological and systemic diseases (47). Despite its profound impact, accurate measurement of its core behavioral expression, namely scratching, and its multidimensional consequences remains methodologically challenging. Critical gaps persist, including the absence of validated tools specifically designed to quantify scratching intensity, limited insight into how this behavior fluctuates with disease activity, and an unmet need for instruments that capture the full impact of scratching on daily functioning within distinct cultural contexts (48). This study aimed to address these gaps through the cross-cultural adaptation and validation of the Scratch Intensity and Impact Scale (SIIS) for Chinese patients with chronic pruritus.

Regarding pruritus intensity, several scales have been validated for chronic pruritus (CP) (49). Among these, the 5-D Itch Scale is a brief, multidimensional instrument whose dimensions are designed to capture various aspects of itch over time, making it a useful outcome measure in clinical trials (19). Although established instruments such as the POEM (50) and Skindex-29 (51) include items assessing itch and sleep, their dependence on single-item measurement constrains their capacity to capture the full spectrum of scratching intensity, its nocturnal variations, or the resulting multidimensional and cascading impact on daytime functioning. Consequently, the creation of a multi-item, fine-grained instrument specifically designed to assess scratching behavior and its repercussions, such as the SIIS, is crucial for advancing both clinical management and mechanistic research in chronic pruritus. This holds particular relevance for nocturnal symptoms, which constitute a pivotal yet under-investigated facet of conditions such as atopic dermatitis and psoriasis. In contrast to single-item measures, the Chinese SIIS, with its multi-item structure, allows for a fine-grained assessment of how nocturnal scratching intensity and its impact on daytime function interrelate, thereby informing targeted management strategies. This study entailed the cross-cultural adaptation and psychometric validation of the SIIS for use in Chinese patients with chronic pruritus. As a patient-reported outcome measure, it is easy to administer and has the potential to empower clinicians and researchers to better quantify and understand the core behavioral experience as well as the resultant burden in patients with chronic pruritic conditions.

The psychometric validation established robust measurement properties for the Chinese SIIS. EFA confirmed the original two-factor structure (scratching intensity and impact on quality of life), thereby supporting its cross-cultural validity. Notably, our analysis revealed a culturally informative enhancement in the factor structure: the two factors accounted for a substantially greater proportion of total variance (79.10% vs. 64.59%) and demonstrated a more balanced contribution compared to the original scale, in which the impact factor was dominant. This enhancement suggests that the scratching intensity dimension may be more distinct and psychometrically robust within the Chinese clinical context, potentially reflecting cultural differences in symptom reporting or disease experience. The exceptionally high item loadings (0.748–0.941) on their intended factors further confirm the excellent internal structure and clarity of the scale’s construct.

Beyond structural validity, our analysis revealed a potentially culturally nuanced pattern in symptom reporting and appraisal. Although total and subscale scores were marginally lower among Chinese patients, they reported a disproportionately greater impact on daily life per unit of scratching intensity. One possible interpretation of this dissociation is that for Chinese patients, a given level of scratching behavior is associated with a disproportionately higher perceived impact on quality of life. This pattern could potentially be understood through the lens of response shift theory (52), which posits that patients adapt their internal standards and values when coping with chronic illness. If such processes were present, two mechanisms could potentially operate simultaneously: a recalibration of the intensity scale (where stoic norms (53) lead to under-reporting of scratching severity) and a reprioritization of values (where the social and relational consequences of scratching are assigned greater importance). Thus, the observed score pattern may reflect a culturally shaped appraisal process, although alternative explanations cannot be ruled out. Several limitations temper this interpretation. The cross-sectional design precludes direct tracking of intra-individual changes in internal standards; unmeasured confounders (e.g., disease phenotype, health literacy) could also contribute to the dissociation. Longitudinal studies that embed a then-test procedure, cognitive-debrief interviews, and differential-item-functioning analyses (54) [as recommended by cross-cultural adaptation guidelines (55)] are therefore needed to quantify the magnitude of each response shift component and to disentangle genuine clinical change from scale recalibration (56). Nonetheless, the data underscore that culture does not act as a passive backdrop; it functions as an active prism that filters sensory experience, re-orders domain salience, and ultimately redefines what “ill-health” means for the patient. Ignoring these culturally driven appraisal shifts risks mis-estimating minimal clinically important differences and may inadvertently penalize normative coping styles as under-reporting. Incorporating RS-sensitive designs into future trials will allow more accurate evaluation of treatment efficacy and foster patient-centered care that respects culturally embedded illness experiences.

A comprehensive psychometric comparison between the original and the culturally adapted SIIS indicates substantial enhancement in the Chinese version. The high Cronbach’s *α* (0.971) and McDonald’s *ω* (0.969) values observed in this study indicate excellent internal consistency but may raise questions about potential item redundancy. To examine whether the scale captures two distinct dimensions or predominantly a single general factor, we compared the fit of the two-factor model against a one-factor model. The two-factor model demonstrated substantially better fit than the one-factor model across all indices (*χ*^2^/df = 2.09 vs. 5.23; CFI = 0.985 vs. 0.892; RMSEA = 0.078 vs. 0.142), The one-factor model consistently failed to meet acceptable fit thresholds, providing strong empirical evidence against a dominant general factor and supporting the two-factor structure. The factor correlation (*r* = 0.722; 52% shared variance) further indicates that the two factors, while substantially associated, retain considerable unique variance (48%), confirming they are related yet distinguishable dimensions. Conceptually, the two subscales capture different aspects of the scratching experience—behavioral intensity versus functional consequences—both of which are clinically meaningful for comprehensive patient assessment. The adapted scale showed superior internal consistency, with Cronbach’s *α* for the total scale (0.971) and its subscales (0.980, 0.962) notably surpassing the high benchmarks established by the original instrument (total *α* = 0.91; Impact *α* = 0.85). This elevation in α values reflects exceptional item clarity and conceptual coherence achieved through the adaptation process. Furthermore, the adapted scale demonstrated a substantial improvement in temporal stability, with an excellent test–retest reliability of 0.958, significantly surpassing the moderate test–retest correlation (*r* = 0.66) reported for the original scale. This improvement stemmed from key methodological refinements: employing a larger retest sample (*n* = 50 vs. *n* = 21), and, crucially, controlling for confounding symptom fluctuations by restricting analysis to clinically stable participants (57). This rigorous design ensures that the high test–retest correlation predominantly reflects the instrument’s measurement stability, rather than extraneous clinical variance. Collectively, these findings demonstrate that the Chinese SIIS not only preserves the core psychometric strengths of its predecessor but also achieves enhanced reliability across both internal consistency and test–retest dimensions. This robust reliability profile establishes a solid foundation for its application in both clinical research and practice within Chinese populations. Convergent validity was supported by a significant positive correlation with the 5-D Itch Scale total score (*r* = 0.591, *p* < 0.001). Beyond this convergent validity, the SIIS proves its unique value by elucidating the specific linkage between nocturnal scratching behavior and its downstream functional impact in daylight hours. This subtle yet critical linkage remains elusive to generic sleep questionnaires or single-item assessments. The capacity of the SIIS establishes it as an essential, fine-grained instrument for informing tailored clinical management in chronic pruritus.

The successful validation of the Chinese SIIS provides a pivotal tool for advancing scientific inquiry and clinical practice in chronic pruritus. From a clinical perspective, its sensitivity to nocturnal symptoms positions it as an optimal primary endpoint in interventional trials aimed at disrupting the debilitating itch-scratch-sleep cycle. From a methodological standpoint, future longitudinal research must utilize this scale to establish its responsiveness to therapeutic interventions. Theoretically, the identified culturally mediated dissociation between reported behavior and perceived burden opens a vital line of inquiry. This inquiry should focus on the specific cognitive and psychosocial mediators, for example illness representations and adaptive coping strategies, that fundamentally shape the appraisal and endurance of physical symptoms. Advancing these research trajectories will not only refine treatment strategies for pruritus but also enrich a more sophisticated, cross-cultural understanding of the pathways through which chronic symptoms erode quality of life.

### Limitations

Several limitations should be considered when interpreting these findings. First, the sample was confined to adult patients (18–70 years) with atopic dermatitis or psoriasis from a single tertiary hospital in Northeast China. While these two conditions represent the most common causes of chronic pruritus in dermatological settings, our results may not be directly generalizable to other pruritus etiologies (e.g., renal, hepatic, or neuropathic pruritus), pediatric or geriatric populations, or patients from other regions with different cultural and healthcare backgrounds. Second, the cross-sectional design and single-center recruitment further limit the external validity of the scale. Future multicenter studies with larger and more diverse samples—including different age groups, pruritus etiologies, and geographic regions—are warranted to confirm the factorial structure, measurement invariance, and normative data of the SIIS-C across various patient subgroups. Third, the inherent subjectivity of self-report measures presents an interpretative challenge. The observed dissociation between scratching intensity and impact scores may reflect authentic cognitive reappraisal (response shift), be confounded by unmeasured biological factors (e.g., distinct symptom phenotypes or disease severity), or represent an amalgam of both. Future multimethod studies that integrate SIIS profiles with objective biosensor data (e.g., actigraphy-derived sleep metrics, digitally quantified scratching episodes) and relevant inflammatory biomarkers are needed to disentangle these possibilities and anchor the scale to external biological referents of disease burden. Furthermore, our interpretation of the observed dissociation between intensity and impact scores is limited by the cross-sectional design. Although this pattern is compatible with response shift and culturally mediated appraisal processes, these mechanisms cannot be directly confirmed without longitudinal designs incorporating then-test procedures or repeated measures. Future studies that integrate longitudinal response-shift methods, measurement invariance testing across cultural groups, and qualitative cognitive-debrief interviews are needed to determine whether these processes actually occur and to quantify their magnitude. Fourth, although we treated the 5-point Likert items as continuous based on established methodological guidelines and employed robust ML (MLR) estimation for CFA, we acknowledge that methods explicitly modeling ordinal data (e.g., polychoric correlations with WLSMV estimation) may be theoretically preferable. However, our supplementary analyses using these ordinal estimation methods yielded highly consistent results with our primary analyses, suggesting that our conclusions are robust to this choice and that treating the data as continuous did not bias our findings. This consistency further strengthens confidence in the robustness of the factor structure. Future studies with larger samples may benefit from employing these approaches to further confirm the stability of the factor structure. Additionally, convergent validity was examined using the 5-D Itch Scale as a reference; while this is a well-validated multidimensional pruritus measure, it is not a gold standard for scratching-specific constructs, and future validation studies may benefit from including additional instruments that more directly assess scratching behavior and its functional impact.

## Conclusion

This study provides evidence for the Chinese SIIS as a psychometrically robust, patient-reported outcome measure for assessing scratching intensity and HRQoL impact in adult patients with chronic pruritus due to atopic dermatitis or psoriasis in dermatology settings. The observed pattern of lower intensity yet higher impact scores points to a culturally mediated response shift, underscoring the need for RS-sensitive interpretation in this population. We suggest its potential utility in clinical practice and research, while acknowledging that further validation in broader chronic pruritus populations (e.g., other etiologies, age groups, and geographic regions) is warranted to inform personalized management that aligns with patients’ lived experience and improves health-related quality of life.

## Data Availability

The raw data supporting the conclusions of this article will be made available by the authors, without undue reservation.
